# Nothing But Tears

**DOI:** 10.3201/eid1505.081412

**Published:** 2009-05

**Authors:** Damian J. Krysan

What do I do
My little one,
Lying oh so low?
Is all that I do
For me or for you,
Hoping for a cure?
Do I take your hand,
Do I let you lie,
Do I leave you alone in the snow?
To do no harm,
To close my bag,
To treat you with nothing but tears.

